# Perspectives of pharmacy staff on provision of self-care and minor ailment education in primary schools: a qualitative study

**DOI:** 10.1007/s11096-024-01753-7

**Published:** 2024-06-11

**Authors:** Samira Osman, Vibhu Paudyal, Zahraa Jalal, Christine Hirsch

**Affiliations:** 1 School of Pharmacy, College of Medical and Dental Sciences, University of Birmingham, Edgbaston, Birmingham, B15 2TT UK; 2 Florence Nightingale Faculty of Nursing, Midwifery and Palliative Care, King’s College London, London, UK

**Keywords:** Children, Education, Minor ailments, Primary schools, Self-care

## Abstract

**Background:**

Pharmacy professionals, given their health expertise, can play a role in enhancing health education within their communities and among patients. The potential of the pharmacy workforce to enhance health education among primary school children is underexplored.

**Aim:**

This study aimed to investigate the perspectives of pharmacy staff on the provision of education regarding self-care and treatment of minor ailments to primary school-aged children and to identify roles that pharmacists could play in this regard.

**Method:**

Qualitative semi-structured interviews were conducted online with frontline pharmacy staff in the UK who had patient facing roles with primary school-aged children and parents. Interviews were audio-recorded and transcribed verbatim. Thematic data analysis was applied to the transcripts.

**Results:**

A total of 17 participants were recruited. Participants included 12 pharmacists, two pharmacy technicians and three pharmacy dispensers. All participants worked within community, hospital or primary care facilities.

Five themes emerged from the data analysis: sources of health knowledge accessed by children and parents; a perceived lack of knowledge regarding self-care and treatments for minor ailments among children and parents; a perceived positive impact of education on self-care; barriers to health education; and the potential role of pharmacy staff in self-care education in schools.

**Conclusion:**

Pharmacy staff recognise the value of integrating health education into primary school curricula. A collaborative approach with educational institutions could bridge the gap in knowledge regarding self-care and treatment of minor ailments, and could empower children and reduce unnecessary use of healthcare resources.

## Impact statements

- This study highlights important gaps in provision of health education in primary schools around self-care and management of minor ailments from the perspectives of pharmacist and pharmacy staff.
- By leveraging the expertise of pharmacists, a comprehensive health education program could be developed, benefiting primary school pupils and their families.
- Policy, procedural and financial barriers could deter future involvement of pharmacist and pharmacy staff in the delivery of health education in school settings; addressing these barriers require multi-sector collaborations.

## Introduction

Pharmacists, given their health expertise, have a potential role in delivering health education within their communities. Pharmacists’ roles in health promotion have been recognised globally [1]. For example, they have been long known as public health practitioners in several countries including the UK, USA and Canada [2, 3]. Pharmacists’ public health roles in the UK include screening of chronic health conditions, advice provision, referral, non-medical prescribing and social prescribing [4–7].

Health literacy, defined as the ability to obtain, understand, evaluate and apply health-related information for informed decision-making [8] is known to impact on health outcomes. Wide disparities in health literacy levels among populations are well known with low levels of health literacy associated with adverse health behaviours and outcomes [9]. Through various public health roles that are embedded in pharmacy services, such as the Healthy Living Pharmacy initiatives [10], pharmacy staff in the UK have the potential to provide health education, improve health literacy and mitigate health inequalities [11]. In addition, pharmacists and their pharmacy staff are trained to advise and support the public on a range of minor ailments such as coughs, colds, headaches and indigestion [12]. They can also provide advice on lifestyle changes and self-care measures that people can take to manage these conditions [13, 14]. Studies have shown that minor ailment schemes delivered by pharmacy teams can promote efficiencies within the healthcare system and lead to cost savings [15]. The UK government has recognised the role of pharmacy teams in the treatment of minor ailments and self-care, and policies have been developed to support and promote this role [16].

There are few published studies on the provision of health education by pharmacy staff to young children. Matson et al. evaluated the impact of a health education programme led by student pharmacists on school children's understanding of health topics in Kingston, USA [17]. The programme covered nutrition, physical activity, summer safety, hygiene, medication safety, and prevention of the use of tobacco. The children’s level of knowledge of health promotion strategies, communication, and the role of the pharmacist in childhood health education increased by 20.4, 15.7 and 1.8%, respectively. This research supports the hypothesis that pharmacists can contribute meaningfully to children’s health education by enhancing knowledge and promoting healthy behaviours [17].

Another study conducted in the USA evaluated a student pharmacist-led programme that was designed to educate children aged 9–11 years on the safety of over-the-counter (OTC) medicines [18]. The programme highlighted that children needed to understand how to handle OTC medicines safely, information about safe dosages and storage of the medication, what constituted an unsafe situation and where they should go for help if they needed it. Suggestions for programme enhancement included parent education. Overall, the initiative was seen as successful in teaching children about safe medication use and offering student pharmacists valuable experiential learning opportunities [18]. There is evidence that pharmacists and pharmacy staff could provide further health education to young children.

### Aim

This study was designed to investigate the perspectives of pharmacy staff on their provision of self-care and minor ailments education to primary school-aged children and to identify roles that pharmacy professionals could play in the future.

### Ethics approval

The University of Birmingham Science, Technology, Engineering and Mathematics Ethical Review Committee gave ethical permission for this study, approval number ERN_21-1091 on 18.10.2021.

## Method

The reporting of this study adheres to the guidelines outlined in the consolidated criteria for reporting qualitative research (COREQ) [19].

Qualitative, semi-structured interviews were conducted with frontline pharmacy staff who had experience interacting with parents and children.

### Participants and recruitment

Snowball sampling was adopted to enhance recruitment to the study. Invitations were sent through the researchers’ professional network of pharmacists in the UK and through social media posts on LinkedIn and X. To be included in the study, participants had to be working in patient-facing roles within any pharmacy sector and have interacted with school-aged children or parents on behalf of children. Any interested pharmacy staff were asked to contact the lead researcher by email. No incentive was offered for participation in the study. Informed consent was obtained from all participants regarding their involvement in the study and the subsequent publication of anonymised data. Participants received written information about the study and consent forms prior to interviews, and verbal consent was obtained at the outset of each interview. No minimum sample size was set; all interested participants were interviewed.

### Data collection

Using the initial literature search and the experience of the study team, an interview plan that comprised a core set of questions was drafted and reviewed. A pilot interview was conducted with one participant, and the findings of this interview were included in the final results. The use of an interview schedule ensured uniformity in questioning, but the asking of open-ended questions in a semi-structured format accommodated flexibility to explore topics that were important to each participant [20].

Interviews were conducted by the lead researcher (SO) via Zoom® between October 2021 and January 2022 on dates that were convenient to each interviewee. They were recorded in audio format. Transcripts were generated by the Zoom software. The use of peer reviews and debriefing within the study team ensured methodological rigour and trustworthiness.

### Analysis

After the interviews, the transcripts were checked for missing information and typographical errors, and were all anonymised. The data were analysed thematically in a flexible way to ensure that the collected data was understood comprehensively [21].

After the initial two interviews, transcripts were reviewed independently by SO and a senior researcher (VP) to generate and compare initial codes. The use of two researchers working independently ensured that the thematic analysis was data-driven [22]. NVivo 12 was used to code the transcripts to specific words and phrases. These codes were then categorised into overarching themes, which formed the basis of the thematic framework that was then applied to all interview transcripts for coding and analysis. Five themes were identified. Data saturation was reached when no new themes emerged from the data.

## Results

### Demographic data

Seventeen participants were interviewed: 12 pharmacists, two pharmacy technicians and three pharmacy dispensers. All the participants worked within community, hospital or primary care facilities and had patient-facing roles that involved interactions with children of primary school age. Most (n = 16) participants practised in England, the one other in Scotland. Interviewees’ levels of experience in their roles ranged from months to over 20 years. No participants withdrew from the study.

Table 1 shows the interviewee demographics.

**Table 1:** Demographics of pharmacy staff included in the study

Participant	Gender	Job role	Sector	Experience
1	Male	Pharmacist	Primary care	15–19 years
2	Female	Pharmacist	Community	1–5 years
3	Female	Dispenser	Community	20 + years
4	Male	Dispenser	Community	6–10 years
5	Female	Pharmacist	Primary care	11–14 years
6	Male	Pharmacist	Community	6–10 years
7	Female	Pharmacist	Community	6–10 years
8	Male	Pharmacy technician	Primary care	1–5 years
9	Female	Pharmacist	Community	6–10 years
10	Female	Pharmacist	Community	11–14 years
11	Male	Pharmacist	Primary care	15–19 years
12	Female	Pharmacist	Community	20 + years
13	Female	Pharmacist	Hospital	6–10 years
14	Female	Pharmacy technician	Community	15–19 years
15	Male	Dispenser	Community	15–19 years
16	Female	Pharmacist	Primary care	11–14 years
17	Male	Pharmacist	Community-Scotland	20 + years

The five overarching themes that emerged from the analysis were: the sources of health knowledge that were used by children and parents; a perceived lack of knowledge regarding self-care and minor ailment treatment in school-aged children; a perceived positive impact of self-care and minor ailment education; barriers to self-care and minor ailment education; and the potential for pharmacists to play a role in school education.

### Sources of health knowledge used by children and parents

Interviewees agreed that social media and the internet seemed to be the usual sources of health-related information for parents of school-aged children. Interviewees were concerned about the quality of information available through these sources and highlighted a need to educate parents and children about reliable sources of information. Interviewees reported that National Health Service (NHS) websites seemed to be widely used by parents for health advice, but that some parents also used Google searches, which could access less reliable information.

*“It depends on which websites you are using, as you find a lot of scare stories on Google, but accredited websites like NHS websites are recommended.”* P12

When the interviewees were asked where children gained health knowledge from, they stated that they understood school, parents and outside care facilitators to be the most common sources. It was apparent that interviewees recognised the potential for schools to play a key role in the promotion of health literacy and the fostering of a culture of well-being among primary school children.

*“Schools play a significant role in the lives of children; children form good relationships with the adults whom can teach them how to care for themselves whilst they are in their care.”* P14

Respondents perceived that generally, parents actively provided health-related information and guidance to their children. They considered that parents were influential in shaping their children's health behaviours, and the interviewees appreciated the collaborative efforts between parents and schools in the promotion of health education.

*“I think so many children gain this type of knowledge regarding self-care from their parents because children spend most time at home.”* P9

Some participants suggested that religious education may contribute to children's health knowledge, as they recognised that religious teachings or practices often incorporated elements of self-care or health-related information. One participant highlighted that religious education could promote holistic well-being and instil health-related values and practices among these children.

*“Many children get this type of knowledge from religious education. To me, self-care is something where you have the knowledge and confidence to deal with it, and you know, when you do need help, where you can ask for help. The potential role of religious education is very much involved.”* P8

### Perceived gap in self-care and minor ailment education in primary school-aged children

Interviewees explained that, in their experience, there was a clear lack of knowledge regarding self-care and minor ailment treatment in families with children of primary school age. Interviewees reported that since the coronavirus pandemic, increased numbers of parents had sought information and advice from community pharmacies regarding common childhood illnesses. The study participants said that many parents required reassurance regarding childhood illnesses, and they considered that a child-friendly education programme to teach parents and children how to manage self-limiting minor ailments would reduce parents’ fear and empower them to self-care during bouts of illness.

*“There’s not very much information on how to recognise and treat specific illnesses; we can warn children of dangers of sepsis or meningitis in a child-friendly way, but the treatment is better aimed at parents.”* P13

A small number of interviewees suggested that certain behavioural challenges, such as resistance, non-compliance or lack of motivation, may contribute to the gap in self-care knowledge. These interviewees stated that it was important to understand and address these behavioural issues in order to enhance the effectiveness of self-care education interventions.

### Perceived positive impact of education regarding self-care and minor ailment treatments

The study participants considered that self-care education would aid in the promotion of healthy living among children. It was suggested that through educational interventions, children could be encouraged to adopt healthy habits, such as eating healthily, taking regular exercise, and practising good hygiene, and that these practices would instil lifelong healthy behaviours.

*“When children are taught to look after themselves- they will lead much healthier lives. A good diet and exercise are essential life skills.” P8*

The economic advantages of self-care education were also described by interviewees, who implied in their answers that healthcare expenditures could be reduced if children were given information and abilities to self-care and manage minor illnesses. Pharmacists emphasised the financial benefits of encouraging self-care behaviours and cutting avoidable costs by reducing the use of unnecessary OTC treatment.

*“Empowering children with self-care knowledge and skills can lead to a reduction in healthcare costs. Equipping them to address minor illnesses independently fosters personal well-being. This underscores the significance of integrating comprehensive self-care education strategies into public health initiatives, ultimately leading to healthier communities and savings."* P11

Interviewees indicated that increased levels of self-care education would lead to a decrease in the number of calls and patient contacts with primary and secondary healthcare for minor ailments. It was also suggested that if people are better equipped to manage their health independently, the need for medical consultations is reduced. The study participants also suggested that improved self-care education would positively impact the doctor-child-patient relationship.

*“When people are confident in what they know and can manage their illnesses, they don’t really need doctors or pharmacists to tell them, this reduces wasted appointments for us in healthcare.” P1*

Interviewees considered that patient empowerment and confidence would be direct benefits of self-care education. The participants suggested that education fostered a sense of personal empowerment that would enable children to participate actively in their own health management, make informed choices and take control of their health.

*“… confident at that age to be able to deal with them [minor illnesses], then they can only build on that confidence in the long-term and pass that confidence on to others around them, I think that's the main benefit.”* P15

### Barriers to the provision of self-care and minor ailment education

The main constraints that the participants considered would affect the ability of pharmacy professionals to offer education on these topics within schools were political factors, such as policies or regulations, which might impact the availability or accessibility of educational resources. There was a concern that procedural barriers might be a challenge to the introduction of this type of educational initiative within schools. Interviewees expressed concern that significant financial barriers would restrict the implementation of new resources and initiatives within pharmacies and schools.

*“Finances are obvious, isn't it? You know, who sets that time aside, who pays and who funds it? And then I imagine if I was a head of a primary school I'd be saying, ‘if I have to; (but) where do I slot that into my curriculum?’”* P17

Reluctance within a community to change perspectives and behaviours was also noted as a barrier. Participants thought that patients’ cultural beliefs, traditions and practices might hinder the acceptance of change. Some patients have long-held expectations that healthcare professionals are the trusted source of advice, and interviewees perceived that some patients would challenge the concept of taking on responsibility for self-care.

Time was recognised as a barrier to the provision of healthcare education outside school, as parents and children have work schedules and commitments that would take priority over time given to self-care education. It was also suggested that schools might find it difficult to find space within their curricula for extra healthcare learning.

*“Parents and teachers don’t have the time to teach children anything more than what they are already doing. Teachers are under pressure with pay and time, parents are under financial pressure, unless this is in the school day and plan or curriculum it will be difficult for them teach it.” P3*

Interviewees voiced concern that currently, parents and teachers lack the level of understanding required to provide the appropriate level of health education for children. There was a risk that an inconsistent or fragmented healthcare education programme might be offered; an example was that sporadic or irregular delivery of educational materials could limit educational outcomes.

*“Lack of knowledge is also a major barrier that we may face; it's how to teach them and what you can teach them at that age, what would be acceptable to teach them, and how much knowledge to give them.”* P4

### Potential role of pharmacists in education

When asked about what roles pharmacists and pharmacy professionals might play in child healthcare education, the participants were unanimous in the view that the pharmacy sector could play a significant role in providing education regarding self-care to schools and the wider community.

*“(The) pharmacy sector plays a pivotal role in self-care education by serving as a trusted source of knowledge and guidance for individuals. Pharmacists are uniquely positioned to provide essential information on medication management, preventive health measures and lifestyle choices.’’* P2

Most participants stated that pharmacies could collaborate with the education sector and deliver education within schools or train teachers to be able to provide this education. Participants considered teachers to be the experts in teaching and pharmacists in healthcare, and they preferred a collaborative approach.

*“Pharmacists can play a vital role by collaborating with schools. Pharmacy's collaboration with the education sector empowers us to either directly deliver education in schools or train teachers to effectively impart this knowledge. It's a collaborative effort that can shape a healthier future."* P7

The participants strongly recommended the promotion of education within community pharmacies. Some suggested that healthy living sections of the pharmacy could be used to target self-care and minor ailment education towards parents and school-age children. Other participants suggested that community pharmacies could run workshops in the pharmacy, schools or community centres to educate the local community.

## Discussion

### Statement of key findings

The study findings show that pharmacy staff believe there is a lack of education regarding self-care and minor ailment treatment within primary schools. The knowledge gap was perceived to impact negatively on the health and well-being of children and families. Study participants highlighted that the gap could be filled through collaborative efforts between pharmacy staff and teachers to provide targeted health education within primary schools.

### Interpretation

In England, young children of primary school age as a group record the highest number of frequent attendances at Accident and Emergency (A&E) departments [23]. The provision of education regarding self-care and minor ailment treatment could shrink the burden on health services and improve health outcomes. Pharmacists possess a strategic advantage for educating the community in various ways due to their knowledge and accessibility, and this workforce should be utilised for health education [24].

Social media and the internet significantly impact levels of health knowledge, as participants stated that parents used these platforms to gather health-related information. It is crucial to understand the impact of social media on children's and parents' health behaviours and the quality of information they receive [25]. Studies have found that social media misinformation is of concern to healthcare professionals [26]. Over 40% of children aged eight to 12 years use social media [27]; therefore it is imperative to ensure that children are educated about reliable sources of health information on digital platforms and taught the risks of misinformation [28].

Participants in this study suggested that political factors, such as policies and regulations, might impact the availability and accessibility of educational resources in schools. Financial barriers, red tape, and cultural beliefs may hinder the introduction of new materials and resources [29]. Community resistance to change is another known barrier [30].

Participants highlighted the importance of children as well as parents acquiring knowledge and skills related to self-care practices to manage minor ailments. This need could be addressed through the development of educational interventions that empower children and parents to take care of their well-being without the need for medical interventions [31]. It would be necessary to tailor educational approaches to the specific needs and preferences of primary school children; pharmacists have recognised that traditional methods of teaching self-care may not effectively engage and empower young learners [32]. There was also the understanding that children may not possess adequate knowledge about self-care practices, and their apprehension or fear of engaging in self-care could hinder their ability to manage minor ailments effectively. Hence there is a need to address these factors to bridge the gap in self-care education [33].

### Study strength and weakness

A significant strength of this study is its focus on a novel area: the role of pharmacy staff in the provision of self-care and minor ailment education to primary school-aged children. This qualitative study employed semi-structured interviews, which enabled an in-depth exploration of participants' perspectives. Data saturation was achieved, which indicated that the study captured a comprehensive range of views.

To enhance trustworthiness, the study followed several practices. Credibility was maintained through peer debriefing and review of the interview transcripts to ensure accuracy [34]. Dependability was strengthened through adherence to a consistent interview plan and the involvement of multiple researchers in the coding and analysis process [35]. The use of thematic analysis added robustness to the interpretation of the data, and COREQ guidelines were followed.

However, this study has some limitations. The sample size (17 participants) was small, and recruitment relied on social media and professional networks, which could have led to selection bias. The geographical scope was primarily the Midlands region of England, which may have affected transferability to other regions or countries. Additionally, the study was focused on pharmacy staff perspective. Exploring the perspectives of other stakeholders, such as teachers, parents or the children themselves would have enriched the data.

Despite these limitations, this study contributes valuable insights into a previously underexplored area and lays the groundwork for future research into the collaborative roles of pharmacists and educators in the promotion of self-care education among primary school children.

### Implications for practice

Most participants in this study believed that the pharmacy sector could collaborate with that of education to deliver knowledge within schools or to train teachers to provide it [36]. Interprofessional work of pharmacy staff with other healthcare professions, such as general practitioners, nurses and healthcare assistants, is a key to the delivery of such knowledge. Pharmacists’ skills can be exploited to provide education to children directly or to work with the education sector to develop a learning plan to be delivered within schools. A collaborative approach is recommended, as teachers are experts in teaching and pharmacists in healthcare. Community pharmacies can also promote education through workshops, healthy living sections, and workshops in pharmacies, schools and community centres.

### Future research

There is a need to develop a pharmacist-led education programme aimed at primary school children. The success of such a programme should be evaluated to measure its effectiveness on health outcomes and increasing knowledge of self-care and minor ailment education. Technology-supported educational interventions that are designed and delivered by pharmacists are likely to engage young people. Given the increasing demands on healthcare staff, including those in pharmacies, pharmacy support staff can contribute to educational interventions, as evidenced by their roles in Healthy Living Pharmacy initiatives [10].

Before such educational interventions are developed, a key point is to understand the perspectives of wider stakeholders such as school and head teachers, policy makers and parents. It would also be interesting to discover the views of children regarding health education and to evaluate their perceptions on the roles of pharmacists.

## Conclusion

The results of this study suggest that pharmacists can play a crucial role in the enhancement of health education among primary school-aged children, especially in self-care and minor ailments. By collaborating with the education sector, pharmacists can fill the current gap in health literacy, and thereby reducing the number of unnecessary healthcare visits and empowering children and parents to manage minor illnesses independently. This collaborative approach offers a practical way to integrate health education into school curricula and promote healthy behaviours from a young age. Future research could explore the effectiveness of pharmacist-led educational programmes and their long-term impact on health outcomes and healthcare costs. This study provides a solid foundation for the development of collaborative educational initiatives that could benefit children, parents and healthcare systems.
